# Case Report: Secondary ulnar overgrowth and elbow incongruity following chronic forelimb disuse in a dog

**DOI:** 10.3389/fvets.2026.1759250

**Published:** 2026-05-04

**Authors:** Tae-Yoon Eom, Gyu-Sung Choi, Mu-Young Kim

**Affiliations:** 1 Department of Veterinary Surgery, College of Veterinary Medicine, Konkuk University, Seoul, Republic of Korea; 2 YOUR Animal Medical Center, Suwon, Republic of Korea

**Keywords:** 3D printing, disuse atrophy, growthplate, Hueter-Volkmann principle, shoulder arthrodesis

## Abstract

Chronic limb disuse during the active growth phase can induce complex secondary deformities, including paradoxical bone overgrowth. A 12-month-old neutered male Toy Poodle presented with non-weight-bearing right forelimb lameness resulting from a chronic shoulder malunion following failed stabilization of a medial luxation. Computed tomography revealed a complete nonunion of the shoulder joint with severe varus deformity, alongside a significant secondary ulnar overgrowth (6.08% elongation compared with the contralateral ulna) that created a functional ‘short-radius’ configuration and elbow incongruity. A combined surgical approach was employed: shoulder arthrodesis was performed utilizing patient-specific 3D-printed osteotomy and reduction guides to ensure precise alignment and fixation at a target angle of 105°, concurrent with a proximal ulnar osteotomy to restore ulnar congruity. Postoperative recovery was rapid, with functional weight-bearing observed at 1 month. At 9 months postoperatively, the dog exhibited normal limb function without lameness, and radiographs confirmed solid arthrodesis and corrected elbow alignment. These findings remained unchanged at 15 months postoperatively. This case highlights chronic disuse as a potential etiology for paradoxical ulnar overgrowth and demonstrates a successful combined surgical strategy for the management of complex, multi-joint limb deformities.

## 1 Introduction

Asynchronous growth between the radius and ulna during development is a significant cause of canine elbow incongruity. This discrepancy, regardless of which bone is relatively longer or shorter, alters joint mechanics, leading to excessive cartilage loading, pain, and the rapid progression of degenerative joint disease (1, 2). Corrective procedures, such as proximal ulnar osteotomy, are often employed to restore joint congruity, reduce abnormal pressures, and alleviate the associated lameness (1–3).

Separately, chronic lameness in one limb can induce significant compensatory weight shifting, leading to secondary musculoskeletal issues in other limbs. It is well-documented that hindlimb dysfunction, for example, can overload the diagonal forelimb, altering gait and potentially predisposing the overloaded limb to injury (4). In cases of end-stage, irreparable joint damage, such as chronic luxations or severe fractures, shoulder arthrodesis is a well-established salvage procedure that provides pain relief and functional limb use (5).

The surgical correction of such complex, chronic deformities has been increasingly aided by 3D printing technology (6). The use of patient-specific osteotomy and fixation guides allows for improved preoperative planning, enhanced surgical accuracy, and reduced operative time, particularly in cases with severe anatomical distortion (6–8).

This case report describes a unique presentation in a dog with a non-functional forelimb resulting from chronic shoulder malunion. The prolonged period of limb disuse during the animal’s growth phase led to a secondary, paradoxical ulnar overgrowth and severe elbow incongruity. We describe the successful simultaneous surgical correction of both pathologies, utilizing a 3D-printed guide for the corrective shoulder arthrodesis and a proximal ulnar osteotomy for the elbow incongruity. This case highlights chronic disuse as a potential etiology for significant ulnar overgrowth and demonstrates a successful combined surgical strategy for a complex, multi-joint limb deformity.

## 2 Case description

A 12-month-old neutered male Toy Poodle (3.15 kg) presented with non-weight-bearing right forelimb lameness and left hindlimb lameness. At 8 months of age, the dog had been diagnosed with medial shoulder luxation and treated with stabilization using two threaded pins. Other possible causes of forelimb lameness, such as brachial plexus injury or infection, were excluded based on imaging and clinical presentation.

**Figure 1:**
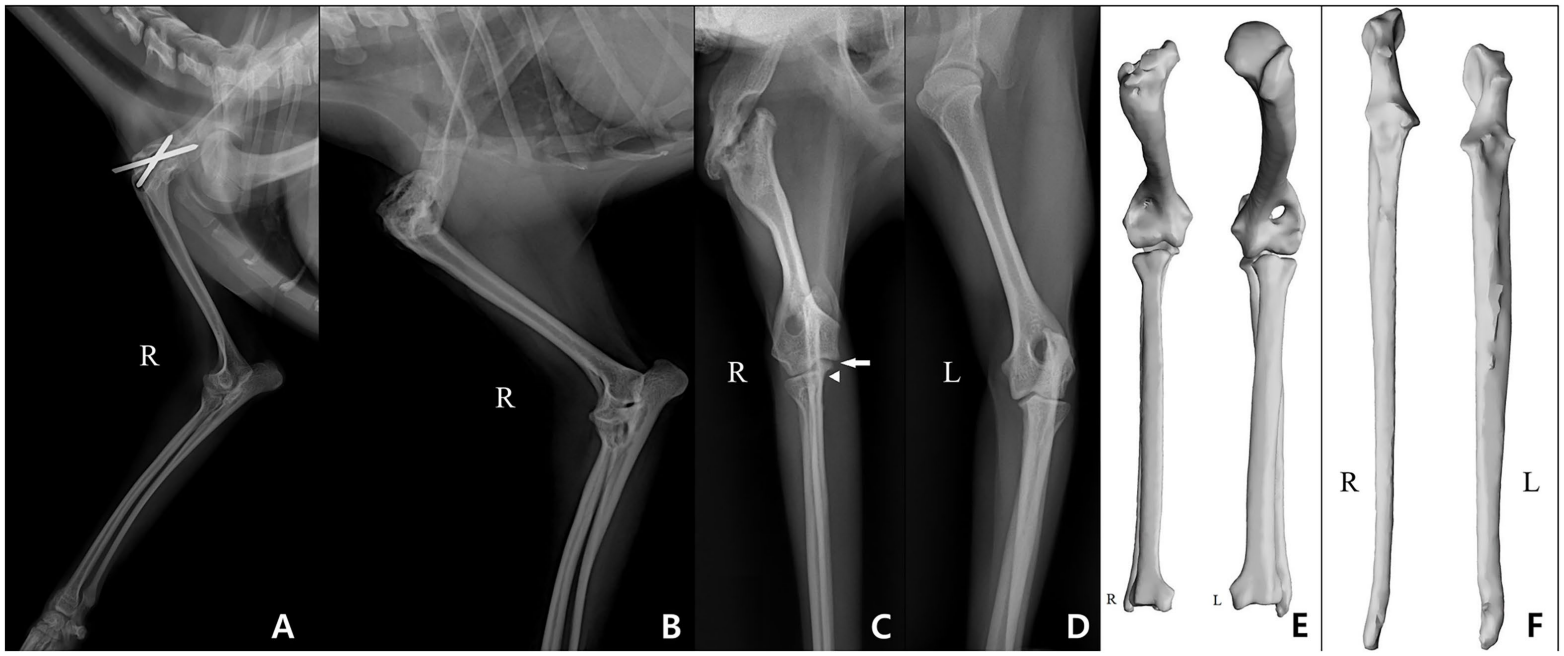
Radiographic and CT evaluation of the forelimbs prior to definitive corrective surgery. Preoperative imaging demonstrates structural abnormalities and joint deformities of the right forelimb. **(A)** Lateral radiograph at initial presentation showing severe malalignment of the shoulder joint with *in-situ* pin fixation. **(B)** Lateral radiograph following pin removal, demonstrating persistent nonunion and abnormal curvature of the humerus and scapula. **(C)** Craniocaudal radiograph of the right forelimb after pin removal, showing varus angulation of the shoulder joint and radioulnar elbow incongruity, characterized by a visible step between the radial head (arrowhead) and the medial coronoid process (arrow). **(D)** Craniocaudal radiograph of the contralateral (left) forelimb, demonstrating normal joint configuration for comparison. **(E)** 3D reconstruction of the scapula, humerus, radius, and ulna of both limbs, showing morphological deformities of the right scapula and humerus, and preserved radial length bilaterally, with marked ulnar overgrowth on the right side. **(F)** 3D reconstruction of the ulna viewed caudally, highlighting abnormal curvature and measurable elongation of the right ulna compared to the left. R and L indicate the right and left forelimbs, respectively.

Lateral radiography of the right forelimb revealed marked medial angulation between the scapula and humerus and visible pin fixation, indicating failed stabilization with varus deformity of the shoulder joint (Figure 1A). The pins were removed, and a femoral head and neck ostectomy was concurrently performed on the left hindlimb, which had been diagnosed with Legg-Calvé-Perthes disease (LCPD). Follow-up radiographs showed persistent nonunion and abnormal joint curvature in the right shoulder, along with radioulnar elbow incongruity (Figures 1B,C). The contralateral limb appeared normal radiographically (Figure 1D). Despite rehabilitation, including passive range of motion (PROM), laser therapy, and high-frequency stimulation, no functional improvement was observed in the right forelimb.

After 1 year of rehabilitation without recovery, surgical intervention was reconsidered. Computed tomography (CT) data was used to generate a segmented 3D bone model. Analysis of this model confirmed the status of the bones and joints, revealing a complete nonunion of the shoulder joint and other pathologies like ulnar overgrowth. Radial lengths were symmetrical (87.0 mm vs. 86.3 mm); however, the right ulna was elongated by 6.3 mm (6.08%), measuring 110.0 mm compared to 103.7 mm on the left (Figures 1E,F). This overgrowth created distinct joint incongruity: while the radial head joint line was level with the lateral coronoid process in the normal left antebrachium, the affected right forelimb exhibited a 3.92 mm discrepancy between these landmarks.

To quantify atrophy, a standardized protocol was established for cross-sectional area (mm^2^) measurements from the segmented 3D bone model. To ensure measurements were comparable and taken from a consistent bone region, landmarks were defined at normalized percentages of total bone length; the 3% proximal and distal landmarks were selected to represent the metaphysis while consistently avoiding the irregular geometry of the articular surfaces, which could skew the area measurements. For the radius, these levels were proximal (3% of total radial length distal from the proximal joint), midshaft (midpoint of total bone length), and distal (3% of total radial length proximal from the distal joint). For the ulna, the levels were proximal (3% of total ulnar length distal from the medial coronoid process), midshaft (midpoint of total bone length), and distal (3% of total ulnar length proximal from the radiocarpal joint). This cross-sectional analysis, based on the bone cross-sectional area at these standardized levels, revealed generalized atrophy on the right, with consistent reductions compared to the contralateral limb (Table 1).

**Table 1:** Comparison of radial and ulnar lengths and cross-sectional areas between left and right antebrachia.

Category	Measurement region	Bone	Right	Left	Δ (mm)	Δ (%)
Length (mm)	–	Radius	87.0	86.3	0.7	0.81%
	–	Ulna	110.0	103.7	6.3	6.08%
Category	Measurement region	Bone	Right	Left	Δ (mm^2^)	Δ (%)
Cross-sectional area (mm^2^)	Proximal	Radius	30.9	47.4	16.5	53.40%
		Ulna	24.5	38.6	14.1	57.55%
	Midshaft	Radius	16.5	22.5	6.0	36.36%
		Ulna	10.6	19.6	9.0	84.91%
	Distal	Radius	10.5	19.6	9.1	86.67%
		Ulna	10.1	11.3	1.2	11.88%

Δ (%) indicates the percentage difference, calculated by dividing the absolute difference (Δ) between the two antebrachia (right and left) by the smaller of the two values.

The dog was diagnosed with chronic shoulder joint nonunion, severe angular deformity, and elbow incongruity secondary to relative ulnar overgrowth. Given the failure of a previous stabilization attempt and the lack of functional improvement after 1 year of rehabilitation, a combined surgical approach was planned. Shoulder arthrodesis was selected as a salvage procedure to address the non-functional, complete nonunion and severe varus deformity of the shoulder. A proximal ulnar osteotomy was planned to correct the elbow incongruity, which was confirmed on CT analysis to be caused by significant relative ulnar overgrowth (6.3 mm). To manage this complex combination of deformities, patient-specific 3D-printed surgical guides and bone models were created from CT data to fit the distorted anatomy, perform rehearsal surgery, and pre-contour the fixation plates.

**Figure 2:**
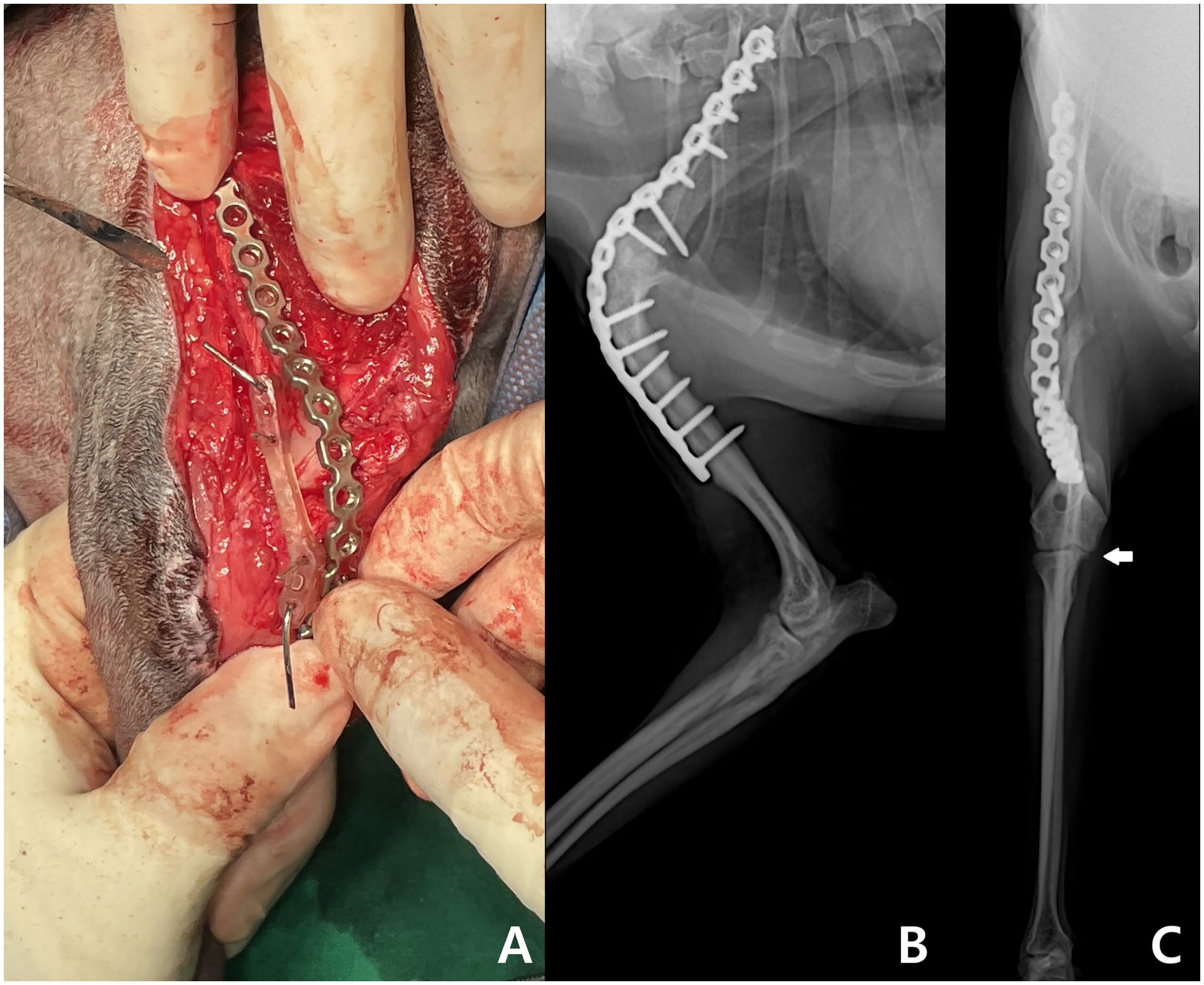
Intraoperative and postoperative images of the forelimbs. **(A)** Intraoperative photograph showing application of a locking plate while the reduction guide is mounted. Lateral **(B)** and craniocaudal **(C)** radiographs of the right forelimb obtained 9 months postoperatively show complete osseous union at the shoulder arthrodesis and ulnar osteotomy sites, along with maintained anatomical alignment and implant stability, indicative of a favorable surgical outcome. The arrow indicates the medial coronoid process, which is aligned with the radial head on the same plane.

Surgery was performed in the left lateral recumbency via a craniolateral approach. Complete nonunion was confirmed intraoperatively by inserting a surgical blade between the scapula and humerus, which separated without resistance. Fibrous tissue interposed between the joint surfaces had tethered the joint in a mal-aligned position and was excised. A 3D-printed osteotomy guide was secured to the scapula and humerus using 1.0 mm Kirschner wires. Osteotomies were made along the guide’s cutting planes. The guide was then removed, and a 3D-printed alignment guide was placed over the Kirschner wires to assist the reduction. With temporary stabilization in corrected alignment, a pre-contoured plate was applied for definitive fixation (Figure 2A). The Kirschner wires were then removed.

The proximal ulnar osteotomy was performed as planned. This was conducted without a patient-specific guide, relying on the surgeon’s intraoperative judgment. A bi-oblique osteotomy was created using an oscillating saw to realign the ulna and reduce abnormal joint pressure. Preoperatively, the forearm showed medial rotational deformity during elbow flexion. After osteotomy, repeated flexion confirmed correction of this deformity, with the forelimb maintaining proper alignment and angulation through the full range of motion. Postoperative management included early PROM, adjunctive laser and hot pack therapy, and regular follow-up visits to support joint flexibility and healing.

Postoperative gait recovery was assessed from surgery to 15 months using a standardized canine lameness grading scale (4). At 2 weeks, the dog intermittently placed the right forelimb on the ground, bearing minimal weight on the toe tips while walking (grade 3 lameness). By 1 month, full weight-bearing was observed during walking and trotting, though the limb was occasionally lifted during galloping (grade 2 lameness). At 2 months, the dog regained functional gait, including galloping. Walking appeared normal; however, during faster gaits, a subtle head lift was intermittently noted on right forelimb contact (grade 1 lameness). At 9 months, the dog showed no lameness during daily activities. Follow-up radiographs confirmed complete recovery without implant loosening or nonunion (Figure 2B) and demonstrated corrected elbow alignment, with the radial head and medial coronoid process on the same plane (Figure 2C). The dog continued to show normal gait and limb function at 15 months postoperatively.

## 3 Discussion

A central and unusual finding in this case was the significant ulnar overgrowth (6.08% elongation compared to the contralateral limb) that developed in the chronically disused forelimb. This occurred despite the radial length remaining symmetrical and the presence of generalized disuse osteopenia, evidenced by markedly reduced cross-sectional area in both the radius and ulna of the affected limb. This presents an apparent paradox: simultaneous bone over-lengthening in the ulna and bone atrophy in the entire antebrachium. This phenomenon can be explained by two distinct biological processes, governed by different mechanical principles, acting concurrently: Wolff’s Law and the Hueter-Volkmann principle (9, 10). Wolff’s Law governs the remodeling of existing bone mass; the absence of mechanical load triggered a potent atrophic signal, leading to the disuse osteopenia seen in both bones (9, 11). In contrast, the Hueter-Volkmann principle governs longitudinal growth at the cartilaginous physes, positing that mechanical compression retards growth, while a reduction in compression accelerates it (10).

The divergent response of the radius and ulna to the same state of disuse is rooted in their fundamentally different biomechanical roles. In a healthy canine forelimb, the radius is the principal axial load-bearing structure, supporting approximately 70–80% of the body’s weight on that limb (12–14). For the radius in this patient, the transition from this high-load state to complete non-use represented a profound physiological shock. This massive change in loading generated two conflicting signals: a physeal command to accelerate growth and a powerful systemic command to atrophy. In this conflict, the atrophic signal was overwhelmingly dominant, effectively suppressing or negating the weaker stimulus for physeal lengthening. The observable result matched our findings perfectly: a bone that became demonstrably thinner but did not overgrow.

The ulna’s experience in the non-use limb was fundamentally different. Its normal role is related more to elbow stability, and it bears only a minimal portion of the axial load (12). Therefore, the transition from this low-load state to a zero-load state was a much less dramatic mechanical change. Consequently, the atrophic signal was significantly weaker than in the radius, allowing the growth-accelerating effects of the Hueter-Volkmann principle to become the dominant response. This effect was then critically amplified by two factors. First, the ulna’s growth is overwhelmingly dominated by its distal physis (85–90% of total growth) (15), which, with its normal compressive load removed, was “unleashed” to proceed at an accelerated rate. Second, a non-use limb is often held in mild flexion, creating a constant, low-grade passive tension in flexor muscles originating on the ulna. This passive tension likely exerted a subtle but continuous distractive force across the distal physis, further stimulating growth (16). This “perfect storm” of reduced compression and passive distraction provides a robust explanation for paradoxical overgrowth.

Although a pre-injury asymmetry of the antebrachium cannot be completely excluded due to the lack of baseline measurements, several observations support the hypothesis that altered mechanical loading contributed to the ulnar overgrowth. The radial lengths were symmetrical between limbs, whereas the ulna demonstrated clear relative elongation compared with the radius, and the deformity developed during a prolonged period of limb disuse while the dog was still undergoing active skeletal growth. These findings suggest that differential physeal loading may have influenced growth modulation consistent with the Hueter–Volkmann principle, although this mechanism cannot be definitively confirmed in a single clinical case.

The severe mismatch in growth—6.08% ulnar elongation versus just 0.81% for the radius—produced a structural alteration of the proximal radioulnar alignment, creating a functional ‘short-radius’ configuration. Unlike the level alignment in the normal left antebrachium, the right forelimb exhibited a 3.92 mm discrepancy. This relative elongation disrupted the normal synchronization of the elbow, causing the radial head motion to lag behind and resulting in abnormally early humeroulnar contact. This asynchronous growth distorted the mechanical axis and was the direct cause of the severe elbow incongruity and varus-like malalignment confirmed intraoperatively (1, 3). Therefore, performing a proximal ulnar osteotomy was as critical as the shoulder arthrodesis to restore joint congruity and overall limb function (2, 3, 5). Furthermore, given the severe nonunion and deformity of the shoulder, the use of 3D printing was invaluable. Virtual surgical planning and patient-specific guides were essential for achieving an accurate osteotomy, stable fixation, and the target arthrodesis angle of approximately 105° (6–8). This technology also allowed for pre-contouring of the plate, which significantly shortened operative time.

Historically, rigid external coaptation, such as a Spica splint, was recommended after shoulder arthrodesis. However, these are associated with significant complications (5, 17). In this case, stable internal fixation was achieved, and a soft padded bandage was used for only 2 weeks postoperatively. This, combined with immediate passive range of motion exercises for the elbow, provided adequate protection without the complications of rigid splinting and led to an excellent functional recovery.

Finally, the patient’s initial presentation involved concurrent left hindlimb LCPD and right forelimb nonunion. LCPD is a developmental condition that causes hindlimb pain (18). It is highly probable that the dog’s early hindlimb lameness from LCPD led to chronic compensatory weight shifting onto the diagonal (right) forelimb (4). This chronic overload may have been a contributing factor to the initial shoulder luxation at 8 months of age. This case highlights the importance of evaluating the entire musculoskeletal system, as compensatory forces can lead to significant secondary pathologies.

This report is subject to the inherent limitations of a single case study. The conclusions drawn, particularly regarding the proposed mechanism of disuse-induced ulnar overgrowth, cannot be generalized to the wider population. The hypothesis that reduced physeal compression (Hueter-Volkmann principle) in the non-load-bearing ulna accelerated its growth, while the radius was primarily affected by atrophic forces (Wolff’s Law), is a compelling interpretation of the clinical data but does not establish a definitive causal relationship. A significant limitation is the lack of histopathological examination. Histology of the distal ulnar physis could have provided direct evidence of its activity, and analysis of the bone tissue could have further characterized the nature of the disuse osteopenia beyond the cross-sectional area measurements.

Genetic factors influencing skeletal development and orthopedic diseases have been increasingly recognized in dogs, particularly in small breeds. Because genetic testing was not performed in this case, a genetic predisposition contributing to asymmetric ulnar growth cannot be completely excluded (19, 20).

Another limitation is the absence of postoperative CT imaging. Although radiographic evaluation confirmed restoration of elbow alignment and successful arthrodesis, CT would have provided a more precise three-dimensional assessment of postoperative anatomical correction.

Finally, at 9 months postoperatively the dog exhibited normal limb function without lameness and radiographs confirmed solid arthrodesis and corrected elbow alignment. At the 15-month follow-up, physical examination confirmed that normal limb function was maintained. Nevertheless, longer-term monitoring would still be necessary to determine whether degenerative changes may develop in the elbow joint or adjacent joints over time.

Therefore, while this case presents a unique and successful outcome, the proposed etiology for the paradoxical ulnar overgrowth remains a strong hypothesis that requires further investigation.

In conclusion, this case report describes the successful management of a complex multi-joint deformity, combining chronic shoulder nonunion with severe elbow incongruity in a young dog. The paradoxical ulnar overgrowth that developed in a limb with profound disuse atrophy highlights a unique potential consequence of chronic non-weight-bearing during an animal’s growth phase. This case demonstrates that a combined surgical strategy, utilizing 3D-printed patient-specific guides for shoulder arthrodesis and a corrective proximal ulnar osteotomy, can successfully address both the primary and secondary pathologies. This approach resulted in the restoration of joint congruity and an excellent long-term functional recovery. This report underscores the importance of evaluating the entire limb for secondary deformities, particularly elbow incongruity, in any case of chronic forelimb lameness.

## Data Availability

The original contributions presented in the study are included in the article/supplementary material, further inquiries can be directed to the corresponding author.
